# Manipulation of the rhizosphere microbial community through application of a new bio-organic fertilizer improves watermelon quality and health

**DOI:** 10.1371/journal.pone.0192967

**Published:** 2018-02-16

**Authors:** Jia Zhao, Jiang Liu, Hong Liang, Jing Huang, Zhe Chen, Yuanjun Nie, Changbiao Wang, Yuguo Wang

**Affiliations:** 1 Biotechnology Research Center, Shanxi Academy of Agricultural Sciences, Taiyuan, Shanxi, China; 2 College of Agriculture, Shanxi Agriculture University, Taigu, Shanxi, China; 3 Institute of Agricultural Resources and Economy, Shanxi Academy of Agricultural Sciences, Taiyuan, Shanxi, China; University of Oklahoma, UNITED STATES

## Abstract

Bio-organic fertilizers (BOFs) combine functional microbes with a suitable substrate and have been shown to effectively suppress soil-borne diseases and promote plant growth. Here, we developed a novel bio-organic fertilizer (BOF) by fermentation of a cow plus chicken manure (M) compost using Fen-liquor Daqu (FLD) as a fermentation starter and compared the compositions of bacterial and fungal communities in the rhizosphere soil of watermelon plants after treatment with different fertilizers. Further, we aimed to explore the mechanisms underlying plant-promoting and disease (*Fusarium* wilt)-suppressing activities of each rhizosphere microbial community. The microbial communities of soil amended with cow plus chicken manure compost (S+M), soil amended with the BOF (S+BOF), and untreated control soil (S) without plants were analyzed through sequence analysis using the Illumina MiSeq platform. The results showed that a new microbial community was formed in the manure compost after fermentation by the Daqu. Application of the BOF to the soil induced remarkable changes in the rhizosphere microbial communities, with increased bacterial diversity and decreased fungal diversity. Most importantly, S+BOF showed the lowest abundance of *Fusarium*. Moreover, watermelon quality was higher (*P* < 0.05) in the S+BOF than in the S+M treatment. Thus, application of the BOF favorably altered the composition of the rhizosphere microbial community, suppressing *Fusarium* wilt disease and promoting plant quality.

## Introduction

In sustainable agriculture, which has been gaining increasing public as well as research interest, the soil is regarded a living system that is significantly affected by the microbial communities present [1]. It is recognized that interactions among soil microbes can establish homeostasis of soil microbial communities, promote plant growth, and suppress soil-borne pathogens [2]. In the rhizosphere, where the soil adheres to plant roots and is impacted by the roots and their exudates, complex biological and ecological processes, particularly plant–microbe interactions, occur [3–6]. This region hosts beneficial microbes as well as soil-borne pathogens, which are in competition [7]. The rhizosphere community contains multiple species that exert beneficial effects on plant growth and health, such as nitrogen-fixing bacteria, mycorrhizal fungi, plant growth-promoting rhizomicrobes (PGPR), biocontrol microbes, and protozoa, while soil-borne pathogens that colonize the rhizosphere cause plant diseases by breaking the protective microbial shield and overcoming the plant’s innate defense mechanisms [8]. The complexity and diversity of microbes in the rhizosphere are essential for maintaining homeostasis in the soil ecosystem [9, 10].

Bio-organic fertilizers (BOFs), which combine functional microbes with a suitable substrate, are more effective than microbes added directly to the soil, and are widely accepted as a promising biological method for suppressing soil-borne pathogens and promoting plant growth [11–13]. Most previous studies have focused on individual specific beneficial microbes, but ignored the global effect of microbial communities [14].

Daqu is a mixed fermentation starter for the most representative light-fragrant Chinese liquor, Fen-liquor. As with other starters used for the brewing of Chinese liquors, the microbial composition of Daqu is extremely complex as the raw material and fermentation environment are not sterile [15]. Most studies use individual beneficial microbes as the fermentation starter, but the use of Daqu to generate fermented fertilizer has not been reported.

The continuous cropping of watermelon is known to lead to notable poor quality, high disease incidence (especially, of *Fusarium* wilt caused by *Fusarium oxysporum* f. sp. *niveum*), and low yield. In this study, we explored the effects of a newly developed BOF, generated by fermenting cow plus chicken manure compost with Daqu, on the interactions between watermelon and rhizosphere microbial communities, as compared to non-fermented cow and chicken manure compost. To this end, we used the MiSeq platform, which allows high-throughput analysis of and deep insight into rhizosphere microbial community composition and diversity [16, 17]. The objectives were to compare the composition of the rhizosphere bacterial and fungal communities after treatment with the different fertilizers and to explore the mechanisms underlying plant-promoting and disease (*Fusarium* wilt)-suppressing activities of each rhizosphere microbial community.

## Materials and methods

### BOF preparation

Fermented cow plus chicken manure compost (1:1, w/w), containing 45% organic matter, 3.1% N, 3.8% P_2_O_5_, and 1.7% K_2_O (Tianli Fertilizer Ltd., Tianli, Yongjishi, China), was used as organic fertilizer (M) (see below). Fenjiu Daqu (provided by Xinhuacun Fenjiu Group Co. Ltd. and prepared from barley and peas in five steps: formulation of ingredients, grinding and mixing, shaping, incubation, and maturation) was inoculated into the fermented cow plus chicken manure compost (1:100, w/w) following the solid fermentation method, and the mixture was fermented for six days at <45°C until it contained 10^9^ cfu g^−1^ dry weight of substrate [18, 19]. Replicate samples of 100 g of fertilizer and mixed starter were collected and sieved through a 2-mm sieve, and stored at −70°C for DNA extraction. Each experiment and treatment was replicated three times.

### Experimental design, treatments, and sampling

Three treatments were designed for sequence analysis, including soil amended with cow plus chicken manure compost (S+M), soil amended with the BOF (S+BOF), and untreated control soil without plants (S). In addition, microbial communities of the cow plus chicken manure compost (M), Fen-liquor Daqu (FLD), BOF and control soil were analyzed to evaluate the inherent microbial communities. A pot experiment was designed to explore the effects of application of the new BOF on continuous cropping of watermelon. Watermelon seeds (cultivar Mini Love) were surface-sterilized in 5% H_2_O_2_ for 30 min to eliminate effects of surface microbes, washed in sterile water three times, and placed in plates covered with sterile wet filter paper for germination at 30°C. Germinated seeds were transferred into a nursery pot (1 seed/pot) containing 100 g of substrate (pure Pindstrup substrate) [20–22]. When they had three true leaves, seedlings with similar growth and height were selected for the pot experiment and transplanted into pots (50 cm high, 40 cm in diameter; 1 seedling/pot) containing approximately 13 kg of fresh soil each. The plants were grown in a greenhouse under ambient conditions (temperature 22–30°C; relative humidity: 92–95%; light intensity: 68–74 klx). The pot soil was collected from a field that had been planted with watermelon the year before (S) and contained 16.59 g kg^−1^ total C, 1.98 g kg^−1^ total N, 21.26 mg kg^−1^ available N, 16.43 mg kg^−1^ available P, and 232 mg kg^−1^ available K, and the pH was 7.08. Two soil treatments were applied: (1) amendment with BOF (80 g kg^−1^, S+BOF), (2) amendment with organic fertilizer (80 g kg^−1^, S+M). Untreated soil without plants (S) was used as the control. The fertilizers were added to and thoroughly mixed with the soil before planting. For each soil treatment we prepared 20 pots, each planted with a single plant. Rhizosphere soil was collected from all 20 plants and pooled together prior to DNA extraction (18). All experiments were reproduced, and represent plants grown from March to May, April to June, and September to November, 2015. In total, per treatment, there were 3 pooled rhizosphere samples used for DNA extractions and 60 plants used for disease index and plant measurements.

### Analysis of disease incidence, disease index, average fruit weight, and soluble solids

*Fusarium* wilt of the watermelon plants was recorded as of 50 days after transplantation, according to typical wilt symptoms. The 20 plants of each replicate were used to calculate the disease index and the percentage of diseased plants, according to the method of Faheem *et al*. using the following equation [23]:

Diseaseindex=∑(A×B)×100/∑B×4,

where A is the disease class (0, 1, 2, 3, 4) and B is the number of plants in the particular disease class. In general, “soluble solids” refers to the carbohydrate content, including sugars and vitamins, of the plant. The higher the content of soluble solids, the better the watermelon quality [24]. Thus, after the growing season, all fruits were collected and their weights were recorded and soluble solids were determined by the Brix spindle method to assess the watermelon quality.

### DNA extraction, PCR amplification, and Illumina sequencing

Genomic DNA was isolated from each soil sample using an E.Z.N.A. Soil DNA Isolation kit (Omega Bio-tek, USA), according to the manufacturer’s instructions [25]. Amplicon libraries were created from the genomic DNA using tagged bacterial and fungal universal primers. Primers 341F (CCTACGGGNGGCWGCAG) and 805R (GACTACHVGGGTATCTAATCC) were used for the V3–V4 region of the bacterial 16S rRNA gene, while primers ITS5F (GGAAGTAAAAGTCGTAACAAGG) and ITS1R (GCTGCGTTCTTCATCGATGC) targeted the ITS1 region of the fungal internal transcribed spacer gene. The reverse PCR primers contained an Illumina adapter and a unique barcode for each sample. The PCR reaction mixtures and thermal profiles were as previously described [18, 26], with some modification. Briefly, 16S rRNA and ITS gene amplifications for each sample were performed in a 50 μl mixture containing 25 μl Master Mix (2×) (New England Biolabs, UK), 200 nM of each primer, 20 ng of template DNA, and nuclease-free water up to 50 μl. The PCR conditions were 94°C for 5 min, followed by 30 cycles of 45 s at 94°C, 30 s at 55°C for 16S or 45 s at 58.5°C for ITS, and 1 min at 72°C, with a final extension of 5 min at 72°C. A pooled sample for sequencing was created by combining equimolar ratios of the amplicons from each sample, followed by gel purification. Samples were quantitated using a Qubit1 2.0. The pooled sample was sequenced on an Illumina MiSeq platform (quality score was Q30 and trim requirement was set at less than 150 bp), at Personalbio Co., Ltd. (Shanghai, China).

### Sequence data analysis

Raw bacterial and fungal sequences were assigned to each sample based on their unique barcodes. The sequences were clustered into operational taxonomic units (OTUs) at 97% sequence similarity using UCLUST [27], after quality control and the removal of chimeras and short, ambiguous, and low-quality sequences using MOTHUR [28]. The OTUs were classified using the Greengenes and UNITE databases (for bacteria and fungi, respectively) [29].

For bacterial and fungal community analysis, the number of sequences of all samples was taken as the lowest number of sequences, based on the smallest number of samples. The “flattening” process (43,806 for 16S rRNA and 25,615 for ITS sequences) was random, and the sequence numbers of all OTUs were resampled to draw rarefaction curves. Rarefaction curves were generated to compare relative levels of OTU richness across all soil samples at an OTU cut-off of 0.03. Alpha diversity indexes, including estimates of richness (Chao1) and the Shannon diversity index [30], were determined by using MOTHUR. To analyze differences in the bacterial and fungal community structures among the samples, partial least squares discriminant analysis (PLSDA) was used [31]. Correlations among the soil treatments (S+BOF and S+M) and microbial phyla were determined by redundancy analysis (RDA) carried out with the “vegan” package of R.

### Statistical analyses

Differences in parameters among treatments were determined using one-way analysis of variance (ANOVA) at the end of each bioassay, followed by Duncan’s multiple range tests, in IBM SPSS Statistics 17.0. A *P*-value < 0.05 was regarded significant.

### Accession number

The bacterial 16S rRNA and fungal ITS sequence data have been deposited in the NCBI sequence read archive (SRA) database under accession numbers SRP107064 (bacterial sequences) and 107117 (fungal sequences).

## Results

### Microbial diversity

The rarefaction curves and the OTU numbers for the bacterial and fungal communities indicated that the data were sufficient for evaluating differences among the treatments, and the fertilizers M, FLD, and BOF had significantly lower diversity (*P*<0.05) than the soil treatments S, S+M, and S+BOF (S1 Fig and S1 Table). Fermentation decreased the microbial diversity in the fertilizer, as indicated by the fact that BOF showed lower bacterial as well as fungal diversity than M. However, when the BOF was applied to the soil (S+BOF), bacterial diversity was increased and fungal diversity was decreased as compared to the S+M treatment (S1 Table).

The estimated richness (Chao1) and Shannon diversity indexes also significantly differed among the treatments (ANOVA, *P* < 0.05). As shown in Table 1, for both bacteria and fungi, the indexes were significantly lower (*P* < 0.05) for M, FLD, and BOF than for S, S+M, and S+BOF. For bacteria, both indexes were higher for BOF than for FLD (*P* = 0.0000; *P* = 0.2409), but lower than for M (*P* = 0.0000; *P* = 0.0142). The indexes were significantly higher for S+BOF than for S+M (*P* = 0.0031;*P* = 0.0038) and S (*P* = 0.0118; *P* = 0.0223). For fungi, both indexes were lower for BOF than for FLD (*P* = 0.5128; *P* = 0.0959) and M (*P* = 0.0047; *P* = 0.0000). However, although the indexes for S+BOF were higher than those for S (*P* = 0.7092; *P* = 0.3482) and lower than those for S+M (*P* = 0.8492; *P* = 0.4970), the differences were insignificant.

**Table 1:** Richness (Chao1) and Shannon diversity indexes for the different treatments. Indexes were calculated based on the OTUs assigned to 16S rRNA and ITS sequences (97% similarity).

Treatment	Bacterial		Fungal	
	Chao1	Shannon	Chao1	Shannon
BOF	1128.813 ± 96.127e	5.432 ± 0.481d	102.093 ± 5.089c	1.887 ± 0.144c
FLD	723.124 ± 78.935d	4.510 ± 0.108d	149.077 ± 25.944c	2.568 ± 0.134c
M	2808.481 ± 40.719c	7.574 ± 0.898c	390.168 ± 93.848b	4.921 ± 0.521b
S	4299.458 ± 53.916b	9.962 ± 0.352b	574.787 ± 29.437a	5.614 ± 0.898ab
S+BOF	4451.099 ± 15.820a	11.922 ± 0.037a	601.394 ± 178.63a	5.982 ± 0.386a
S+M	4254.919 ± 56.699b	9.137 ± 1.963bc	614.927 ± 37.254a	6.246 ± 0.115a

Data are the mean ± standard error (n = 3) and within each column, different letters indicate significant differences (ANOVA; *P* < 0.05; Duncan’s test).

### Microbial community composition

**Fig 1:**
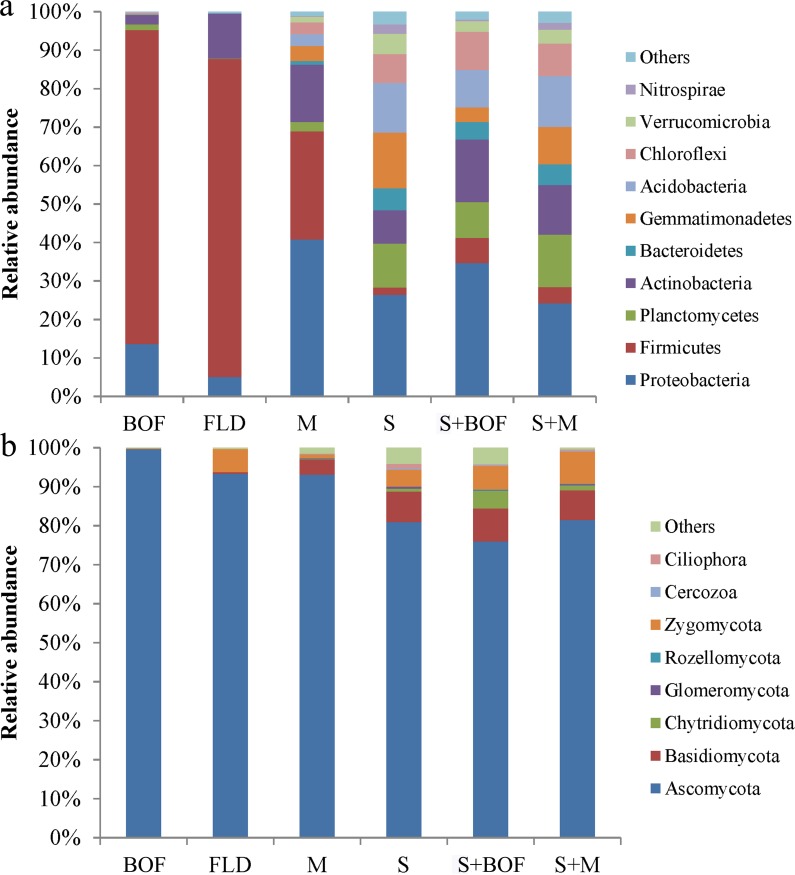
Relative abundances of bacterial (a) and fungal (b) phyla under each treatment.

The dominant phyla associated with the bacterial and fungal communities varied among the fertilizer and soil treatments. The 10 most dominant bacterial phyla identified across all samples were Proteobacteria, Firmicutes, Planctomycetes, Actinobacteria, Bacteroidetes, Gemmatimonadetes, Acidobacteria, Chloroflexi, Verrucomicrobia, and Nitrospirae (Fig 1A). Among these phyla, in fertilizers BOF and FLD, the relative abundance of Firmicutes was greater, while that of Proteobacteria was greater in M. The microbial community was changed by addition of fertilizer to the soil (Fig 1A and S2 Table). As for the soil treatments, Proteobacteria were more abundant than other phyla and while the abundance of Firmicutes decreased significantly. In addition, the relative abundances of Actinobacteria, Chloroflexi, and Planctomycetes were also increased.

With regard to fungi, Chytridiomycota, Zygomycota, Ascomycota, Ciliophora, Basidiomycota, Cercozoa, Glomeromycota, and Rozellomycota were the dominant phyla across all samples (Fig 1B). In the fertilizer, Ascomycota were the most abundant. However, the relative abundance of Ascomycota was lower in soil treatments, but that of Chytridiomycota was higher in S+BOF than in the S (*P* = 0.0000) and S+M (*P* = 0.0000) (S3 Table).

**Fig 2:**
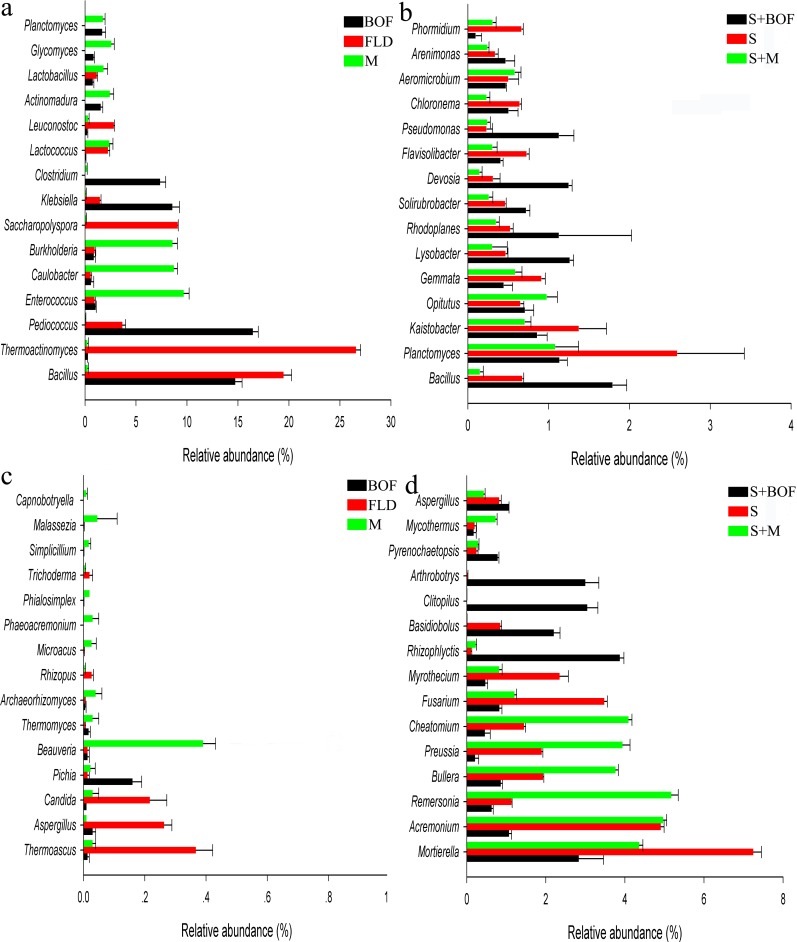
Relative abundances of the 15 most dominant bacterial (a, b) and fungal (c, d) genera.

At the bacterial genus level, comparison of the relative abundances of the 15 most identified genera showed significant differences among fertilizer and soil treatments. Among the fertilizer treatments, FLD had the highest relative abundances of *Thermoactinomyces*, *Bacillus*, *Saccharopolyspora*, and *Leuconostoc* (*P* < 0.05; Fig 2A). The BOF bacterial community was enriched in *Bacillus*, *Pediococcus*, *Klebsiella*, and *Clostridium*, and depleted in *Enterococcus*, *Caulobacter*, *Burkholderia*, *Lactococcus*, *Actinomadura*, *Lactobacillus*, and *Glycomyces* as compared to that in M. However, the BOF-amended soil (S+BOF) had higher abundances of *Bacillus*, *Lysobacter*, *Rhodoplanes*, *Solirubrobacter*, *Devosia*, *Pseudomonas*, and *Arenimona* and lower abundances of *Gemmata* and *Phormidium* than the other soils (*P* < 0.05; Fig 2B).

As for fungi, among the fertilizers, FLD showed the highest relative abundances for the genera *Thermoascus*, *Aspergillus*, *Candida*, and *Rhizopus*, while fermentation enriched the mixed manure compost for the genera *Aspergillus* and *Pichia* and depleted it in *Thermoascus*, *Candida*, *Beauveria*, *Thermomyces*, *Archaeorhizomyces*, *Microascus*, *Phaeoacremonium*, *Phialosimplex*, *Simplicillium*, *Malassezia*, and *Capnobotryella* (Fig 2C). S+BOF was enriched in *Rhizophlyctis*, *Basidiobolus*, *Clitopilus*, *Arthrobotrys*, *Pyrenochaetopsis*, and *Aspergillus* and depleted in *Mortierella*, *Acremonium*, *Remersonia*, *Bullera*, *Preussia*, *Chaetomium*, *Myrothecium*, *Fusarium*, *Pseudogymnoascus*, *Zopfiella*, and *Candida* as compared to the other soil treatments (Fig 2D). Most importantly, the abundance of *Fusarium* was significantly lower (*P* < 0.05) in the soils treated with the BOF (S+BOF).

### Microbial community structure

**Fig 3:**
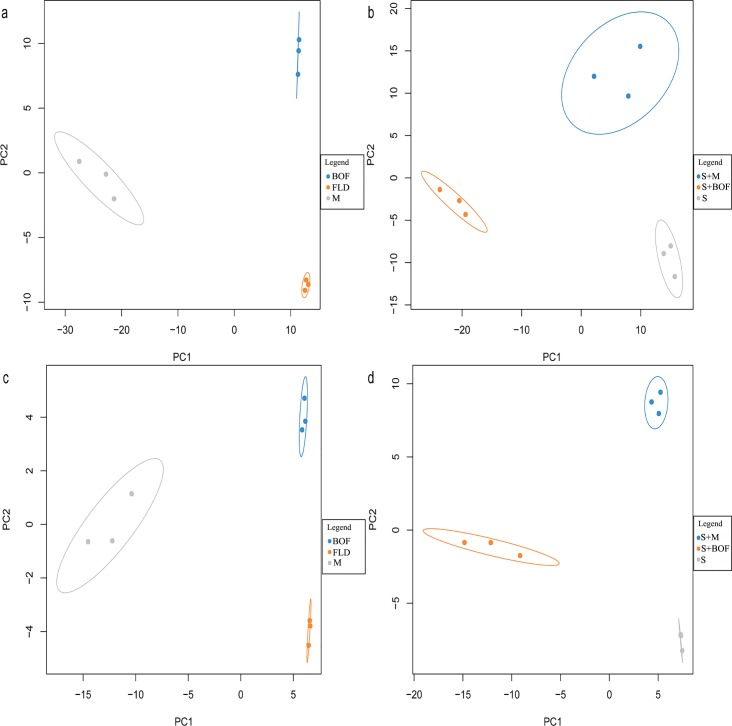
PLSDA of the bacterial (a, b) and fungal (c, d) communities under the different treatments.

PLSDA indicated that the microbial community structure was distinct among the different treatments. For bacteria, among the fertilizers, the BOF treatment was separated obviously from the M and FLD treatments along the second component (PC2) (Fig 3A). M was separated from FLD along the first component (PC1). Among the soil treatments, S+BOF was separated obviously from S+M and S along the first component (PC1), while S+M was separated from S along the second component (PC2) (Fig 3B). For the fungal communities (Fig 3C and 3D), similar results were observed.

**Fig 4:**
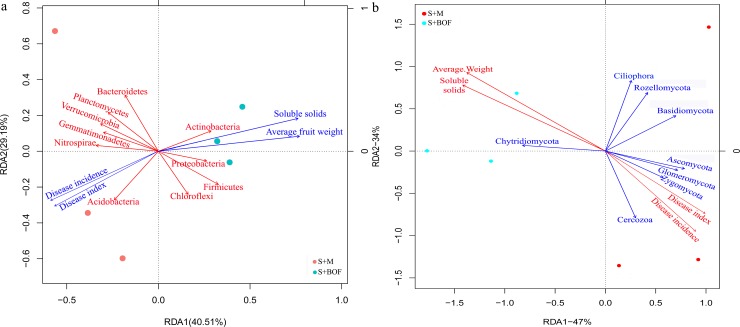
RDA of bacterial (a) and fungal (b) phyla for individual samples of soils treated with the newly developed BOF (S+BOF; blue) and cow plus chicken manure compost (S+M; red).

Relationships among the soil treatments (S+BOF and S+M), watermelon properties, and microbial phyla were analyzed by RDA. For the bacteria, the first two components of RDA explained 40.51% and 29.19% of the total variation (Fig 4A). For the fungi, the first two components of RDA explained 47% and 34% of the total variation (Fig 4B). The first component (RDA1) separated S+BOF from S+M for both bacteria and fungi. Moreover, the relative abundances of the phyla Firmicutes, Actinobacteria, and Chytridiomycota were positively correlated with average fruit weight and fruit soluble solids, and were negatively correlated with the *Fusarium* wilt disease incidence and disease index. For Ascomycota, opposite results were observed.

### Watermelon quality and disease

Average fruit weight and fruit soluble solids significantly differed among the treatments. Average fruit weight and soluble solids in the S+BOF treatment were 2.52 kg and 12.1%, respectively, which were significantly higher (*P*<0.05) than those in the S+M treatment (Table 2). Moreover, the *Fusarium* wilt disease incidence and disease index in the S+BOF treatment were significantly lower (*P* = 0.0213; *P* = 0.0046) than those in the S+M treatment. Thus, the S+BOF treatment was the most effective in reducing the incidence of *Fusarium* wilt and improving watermelon quality.

**Table 2:** Analysis of disease incidence, disease index, average fruit weight, and soluble solids. Pot soils were treated with BOF (S+BOF) or manure compost (S+M).

Treatment	Disease incidence (%)	Disease index	Average fruit weight (kg)	Soluble solids (%)
**S+M**	20 ± 8.67a	43.03 ± 8.69a	2.30 ± 0.03b	10.9 ± 0.3b
**S+BOF**	6.67 ± 2.89b	25 ± 0b	2.52 ± 0.03a	12.1 ± 0.4a

Data are the mean ± standard error (n = 3) and within each column, different letters indicate significant differences (ANOVA; *P* < 0.05; Duncan’s test).

In addition, at the phylum level, the higher abundance of Firmicutes upon S+BOF treatment indicated that the abundance of Firmicutes might be negatively correlated with *Fusarium* wilt incidence and positively with plant growth in watermelon (Fig 1A and S4 Table). High Actinobacterial abundance was also positively correlated with disease suppression, in accordance with a study that showed that Actinobacteria suppress disease owing to their ability to produce an arsenal of compounds, especially antibiotics that promote plant growth and suppress a wide range of soil-dwelling plant pathogens [23]. Ascomycota were more abundant in the BOF than in other fertilizers, but less abundant in S+BOF than in other soil treatments (S).

At the genus level, soil amended with BOF showed the highest abundances of *Bacillus*, *Lysobacter*, and *Pseudomonas* and the lowest abundance of *Fusarium*, while soil amended with the manure compost showed the highest abundance of *Fusarium*. In addition, *Bacillus*, *Lysobacter*, *Pseudomonas*, *Rhizophlyctis*, *Basidiobolus* and *Clitopilus* showed a positive relationship with watermelon yield/quality and a negative relationship with disease incidence/index (S5 Table). These results suggested that a new balance appears in the soil after interaction with the microbial community, and this new balance correlated with plant disease resistance.

## Discussion

In our previous study, we found that continuous cropping of watermelon induces remarkably poor growth, high disease incidence, and low yield [22]. While biological control using microbes is an effective and environmentally friendly strategy for controlling soil-borne fungal pathogens and promoting growth [32, 33], most studies have focused on individual microbial species, ignoring overall effects of microbial communities [14]. In the current study, we applied Daqu-fermented BOF to soil with the aim to alter the microbial community to promote watermelon growth and control *Fusarium* wilt under continuous cropping.

BOFs, which are mixtures of organic fertilizer with beneficial microbes, have been shown to provide an effective tactic for suppressing soil-borne fungal diseases in various crops [34]. In our study, the newly developed BOF was more effective than the organic fertilizer alone (S+M) in reducing the *Fusarium* wilt disease incidence and improving fruit weight and soluble solid content of watermelon. In accordance with these result, previous studies have reported that amendment of suitable substrates and biocontrol agents significantly suppresses soil-borne diseases [16, 22] and have shown that BOF has great plant growth-promoting potential [21, 35]. For example, a novel bioorganic fertilizer significantly (*P* < 0.05) changed the microbial community, reduced disease incidence, and increased fruit yield in tomato [34]. A bioorganic fertilizer consisting of organic fertilizer and *Paenibacillus polymyxa* SQR-21 significantly suppressed *Fusarium* wilt and promoted plant growth in watermelon [36]. A bio-organic fertilizer containing *Trichoderma harzianum* SQR-T037 not only changed the microbial community, but also reduced *Fusarium* wilt in cucumber [37]. A bio-organic fertilizer containing *Bacillus amyloliquefaciens* LH23 or *Bacillus subtilis* LH36 bio-organic fertilizer suppressed bacterial wilt and increased yield in potato [38].

In addition, soil microbial diversity is a key factor influencing soil health and quality. Agricultural treatments can affect soil microbial diversity and even plant health [39, 40]. For each environment, there likely is an optimum soil microbial community that promotes plant growth and protection from disease [41]. This was confirmed in this study (Table 1). The BOF contained bacterial and fungal communities clearly distinct from those of the manure compost, as indicated by the estimated richness (Chao1) and Shannon diversity indexes. This result suggested that a new microbial community had formed in the BOF after fermentation by the Daqu. Moreover, the BOF induced a remarkable change in the rhizosphere microbial communities upon application to the soil. After the growing season, the bacterial and fungal communities clearly differed between the S+BOF and S+M treatments. Our results indicated that bacterial diversity is positively correlated with plant growth and disease suppression, while fungal diversity seems to be negatively correlated with these parameters (S6 Table and Tables 1 and 2). These findings were consistent with those of previous studies that indicated that high bacterial and low fungal diversity are associated with soil-borne disease suppression [12, 16]. This phenomenon may be due to competition for substrates between bacterial and fungal groups or the excretion of antagonistic compounds by bacteria [42]. In summary, BOF application significantly shifted the composition of the watermelon rhizosphere microbial communities by increasing the bacterial diversity and decreasing the fungal diversity.

The results of PLSDA revealed differences in microbial community between the various organic fertilizers, which resulted in differences in microbial community between treatments and control as well as among treatments. This result further indicated that different fertilizers differentially modulate the soil microbial community structure [43, 44]. To understand the relationship between microbial community structure, treatment, and environment, RDA was used. The results indicated that amending the soil with the BOF improved the microbial community structure, plant disease, and watermelon yield and quality, as compared to the other treatments. This finding was similar to those of other studies; for example, *Paenibacillus polymyxa* SQR21 BOF was found to control *Fusarium* wilt in watermelon by suppressing the population of *F*. *oxysporum* and by changing the fungal community structure in the rhizosphere soil [18], bio-fertilizer application altered the composition of rhizosphere microbial community and suppressed *Fusarium* wilt in banana [45], and consecutive applications of BOF effectively suppressed *Fusarium* wilt in watermelon by regulating rhizosphere bacterial diversity [22]. Additionally, beneficial bacteria, such as *Bacillus*, *Lysobacter*, and *Pseudomonas*, were not only much more abundant in S+BOF (Fig 1B), they also showed a positive relationship with watermelon quality and a negative relationship with disease (S5 Table). Similar results were found for *Rhizophlyctis*, *Basidiobolus*, and *Clitopilus* (Fig 1D and S5 Table). These results indicated that improving the plant rhizosphere microbiota has a certain practical significance for controlling soil-borne diseases and promoting plant growth.

In conclusion, the present study demonstrated that the Fen-liquor Daqu, as a mixed fermentation starter, changes the microbial community composition and abundance of manure compost via re-fermentation. The amendment of soils with this new BOF can effectively promote plant growth and suppress *Fusarium* wilt disease in watermelon. This may be owing to the control of the rhizosphere microbial community composition through enrichment of bacterial diversity and depletion of fungal diversity, reduction of *Fusarium* abundance, and stimulation of potentially beneficial taxa such as *Bacillus*, *Lysobacter*, and *Pseudomonas*. Similar results have been reported for watermelon [22], cucumber [46], banana [45], and wheat [47]. Thus, this strategy that changes the microbial community composition of the plant rhizosphere has good potential in controlling soil-borne diseases and improving plant growth.

## Supporting information

S1 Fig Bacterial and fungal diversities under different treatments. Rarefaction curves for bacteria (a) and fungi (b) at 97% similarity. Fertilizer treatments included cow and chicken manure compost (M), Fen-liquor Daqu (FLD), and the new BOF. Soil treatments included untreated soil (S), soil amended with cow and chicken manure compost (S+M), and soil amended with the new BOF (S+BOF).
(TIF)

S1 Table The OTUs number of different treatments of bacteria and fungi. (DOC)

S2 Table Bacterial phylum relative abundance of different fertilizer and soil treatment. (DOC)

S3 Table Fungal phylum relative abundance of different fertilizer and soil treatment. (DOC)

S4 Table The correlation between different microbial phylum and watermelon quality and disease. (DOC)

S5 Table The correlation between different microbial genus and watermelon quality and disease. (DOC)

S6 Table The correlation between microbial diversity and watermelon quality and disease. (DOC)
