# Supplementary figures and images for: Manipulation of the rhizosphere microbial community through application of a new bio-organic fertilizer improves watermelon quality and health
Source: PLoS One. 2018 Feb 16;13(2):e0192967. doi: 10.1371/journal.pone.0192967 (PMC5815603; doi:10.1371/journal.pone.0192967)

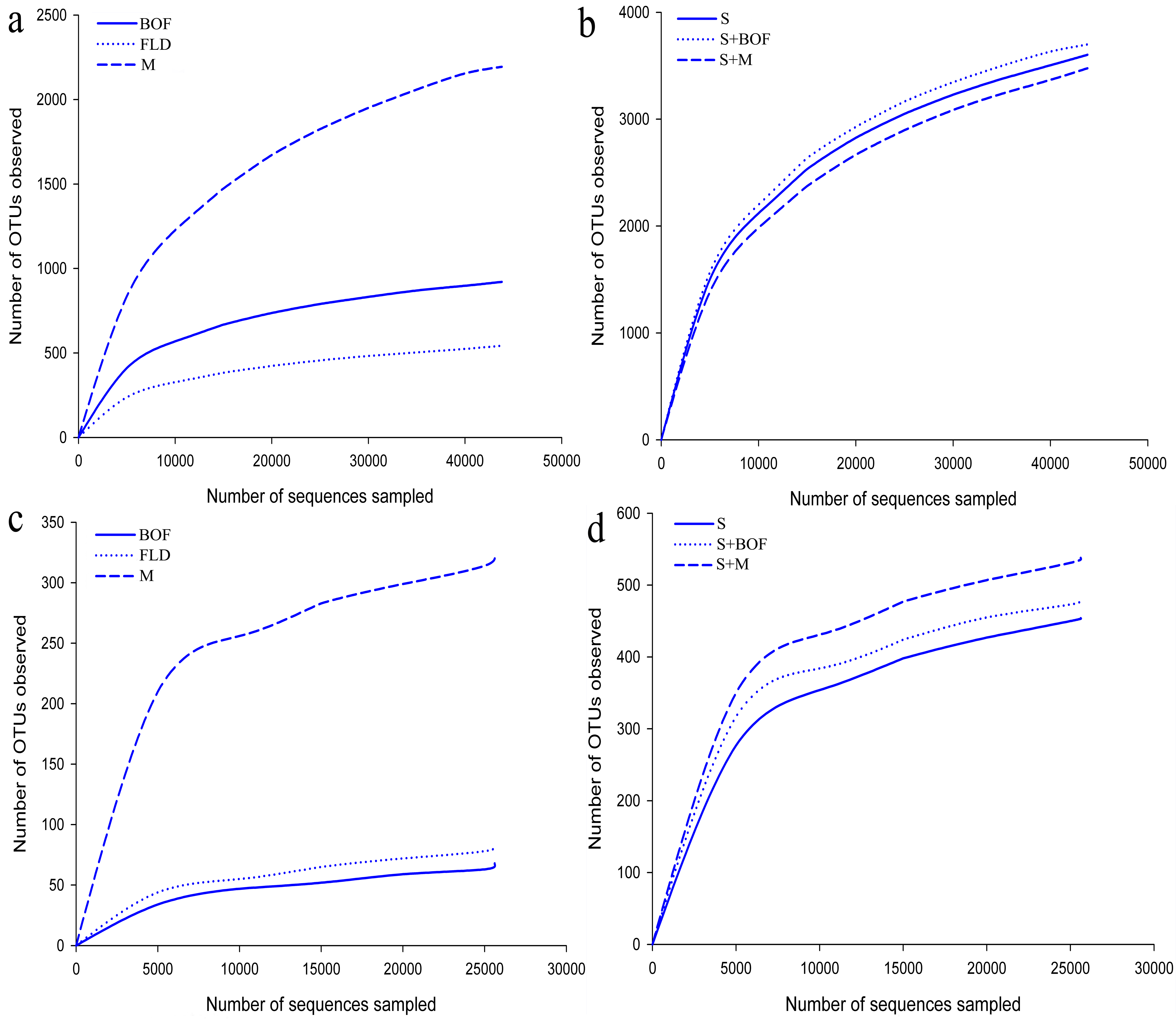

Supplement: S1 Fig — Rarefaction curves for bacteria (a) and fungi (b) at 97% similarity. Fertilizer treatments included cow and chicken manure compost (M), Fen-liquor Daqu (FLD), and the new BOF. Soil treatments included untreated soil (S), soil amended with cow and chicken manure compost (S+M), and soil amended with the new BOF (S+BOF). (TIF) [file pone.0192967.s001.tif]
