# Supplementary material for: Manipulation of the rhizosphere microbial community through application of a new bio-organic fertilizer improves watermelon quality and health
Source: PLoS One. 2018 Feb 16;13(2):e0192967. doi: 10.1371/journal.pone.0192967 (PMC5815603; doi:10.1371/journal.pone.0192967)
Supplement: S1 Table — (DOC) [file pone.0192967.s002.doc]

**S1 Table The OTUs number of different treatments of bacteria and fungi.**

| Microbial | B | FLD | M | S | S+B | S+M |
| --- | --- | --- | --- | --- | --- | --- |
| Bacteria | 1021±15d | 542±15e | 2270±180c | 3766±7b | 3927±92a | 3657±12b |
| Fungi | 75±5e | 90±4e | 331±35d | 531±9c | 566±8b | 634±5a |

Note: Data are the mean ± standard error (n = 3) and within each column, different letters indicate significant differences (ANOVA; P < 0.05; Duncan’s test).
