# Supplementary material for: Manipulation of the rhizosphere microbial community through application of a new bio-organic fertilizer improves watermelon quality and health
Source: PLoS One. 2018 Feb 16;13(2):e0192967. doi: 10.1371/journal.pone.0192967 (PMC5815603; doi:10.1371/journal.pone.0192967)
Supplement: S2 Table — (DOC) [file pone.0192967.s003.doc]

**S2 Table Bacterial phylum relative abundance of different fertilizer and soil treatment.**

| Bacterial phylum | B | FLD | M | S | S+B | S+M |
| --- | --- | --- | --- | --- | --- | --- |
| Proteobacteria | 13.64±0.13d | 5.10±0.20e | 40.34±1.14a | 26.17±2.58c | 34.93±1.77b | 24.06±1.96c |
| Firmicutes | 81.23±1.07a | 81.99±0.81a | 28.16±0.51b | 2.09±0.51e | 6.41±0.33c | 4.40±0.65d |
| Planctomycetes | 1.45±0.09d | 0.09±0.01e | 2.42±0.19d | 11.51±0.49b | 9.18±1.24c | 13.48±0.84a |
| Actinobacteria | 2.60±0.26e | 12.27±0.60c | 14.80±0.23b | 8.95±0.52d | 16.27±0.97a | 12.95±0.94c |
| Bacteroidetes | 0.17±0.12d | 0.00±0.00d | 0.93±0.12c | 5.70±0.29a | 4.55±0.41b | 5.41±0.47a |
| Gemmatimonadetes | 0.10±0.00d | 0.00±0.00d | 3.96±0.08c | 14.29±0.96a | 3.58±0.23c | 9.83±0.52b |
| Acidobacteria | 0.03±0.02d | 0.00±0.00d | 3.18±0.41c | 12.60±1.19a | 9.65±0.54b | 12.88±1.52a |
| Chloroflexi | 0.43±0.08e | 0.00±0.00e | 3.14±0.20d | 7.55±0.59c | 9.96±0.88a | 8.86±0.38b |
| Verrucomicrobia | 0.00±0.00e | 0.00±0.00e | 1.46±0.09d | 5.39±0.39a | 2.75±0.12c | 3.63±0.36b |
| Nitrospirae | 0.06±0.02c | 0.00±0.00c | 0.24±0.03c | 2.49±0.32a | 0.43±0.07c | 1.61±0.50b |
| Others | 0.36±0.45c | 0.67±0.31c | 1.37±0.29bc | 3.25±1.60a | 2.29±0.64ab | 2.88±0.89ab |

Note: Data are the mean ± standard error (n = 3) and within each column, different letters indicate significant differences (ANOVA; P < 0.05; Duncan’s test).
