# Supplementary material for: Manipulation of the rhizosphere microbial community through application of a new bio-organic fertilizer improves watermelon quality and health
Source: PLoS One. 2018 Feb 16;13(2):e0192967. doi: 10.1371/journal.pone.0192967 (PMC5815603; doi:10.1371/journal.pone.0192967)
Supplement: S3 Table — (DOC) [file pone.0192967.s004.doc]

**S3 Table Fungal phylum relative abundance of different fertilizer and soil treatment.**

| Fungal phylum | B | FLD | M | S | S+B | S+M |
| --- | --- | --- | --- | --- | --- | --- |
| Ascomycota | 99.47±0.21a | 93.20±1.95b | 93.23±0.55b | 81.18±2.24c | 75.88±1.46d | 81.68±1.89c |
| Basidiomycota | 0.00±0.00c | 0.60±0.26c | 3.80±0.46b | 7.54±0.98a | 8.63±0.97a | 8.13±0.42a |
| Chytridiomycota | 0.00±0.00d | 0.00±0.00d | 0.10±0.03d | 0.60±0.10c | 4.61±0.63a | 1.35±0.18b |
| Glomeromycota | 0.00±0.00c | 0.00±0.00c | 0.15±0.05bc | 0.50±0.10a | 0.14±0.08bc | 0.30±0.17b |
| Rozellomycota | 0.00±0.00b | 0.00±0.00b | 0.10±0.00a | 0.00±0.00b | 0.12±0.03a | 0.11±0.04a |
| Zygomycota | 0.30±0.17c | 5.80±1.41ab | 0.97±0.06c | 4.70±1.08b | 6.23±0.86ab | 7.28±1.64a |
| Cercozoa | 0.00±0.00b | 0.00±0.00b | 0.10±0.00b | 0.45±0.19a | 0.13±0.06 | 0.30±0.10a |
| Ciliophora | 0.00±0.00c | 0.00±0.00c | 0.10±0.00bc | 1.48±0.47a | 0.15±0.09bc | 0.43±0.12b |
| Others | 0.23±0.15c | 0.40±0.36c | 1.48±0.03b | 3.55±0.55a | 4.11±0.23a | 0.40±0.38c |

Note: Data are the mean ± standard error (n = 3) and within each column, different letters indicate significant differences (ANOVA; P < 0.05; Duncan’s test).
