# Supplementary material for: Manipulation of the rhizosphere microbial community through application of a new bio-organic fertilizer improves watermelon quality and health
Source: PLoS One. 2018 Feb 16;13(2):e0192967. doi: 10.1371/journal.pone.0192967 (PMC5815603; doi:10.1371/journal.pone.0192967)
Supplement: S4 Table — (DOC) [file pone.0192967.s005.doc]

**S4 Table The correlation between different microbial phylum and watermelon quality and disease.**

| **Microbial phylum** | **Disease incidence** | **Disease index** | **Average fruit weight** | **Soluble solids** |
| --- | --- | --- | --- | --- |
| Proteobacteria | -0.56 | -0.49 | 0.8 | 0.76 |
| Firmicutes | -0.68 | -0.58 | 0.76 | 0.79 |
| Planctomycetes | 0.78 | 0.74 | -0.8 | -0.86 |
| Actinobacteria | -0.44 | -0.3 | 0.86 | 0.69 |
| Bacteroidetes | 0.75 | 0.77 | -0.63 | -0.73 |
| Gemmatimonadetes | 0.71 | 0.62 | -0.92 | -0.86 |
| Acidobacteria | 0.41 | 0.27 | -0.81 | -0.69 |
| Chloroflexi | -0.51 | -0.37 | 0.84 | 0.61 |
| Verrucomicrobia | 0.42 | 0.29 | -0.82 | -0.68 |
| Nitrospirae | 0.51 | 0.38 | -0.81 | -0.73 |
| Ascomycota | 0.79 | 0.75 | -0.77 | -0.71 |
| Basidiomycota | -0.58 | -0.62 | 0.23 | 0.58 |
| Chytridiomycota | 0.19 | 0.04 | -0.49 | -0.36 |
