# Supplementary material for: Manipulation of the rhizosphere microbial community through application of a new bio-organic fertilizer improves watermelon quality and health
Source: PLoS One. 2018 Feb 16;13(2):e0192967. doi: 10.1371/journal.pone.0192967 (PMC5815603; doi:10.1371/journal.pone.0192967)
Supplement: S5 Table — (DOC) [file pone.0192967.s006.doc]

**S5 Table The correlation between different microbial genus and watermelon quality and disease.**

| **Microbial genus** | **Disease incidence** | **Disease index** | **Average fruit weight** | **Soluble solids** |
| --- | --- | --- | --- | --- |
| Bacillus | -0.83 | -0.89 | 0.95 | 0.88 |
| Planctomyces | 0.23 | 0.16 | -0.04 | -0.02 |
| Lysobacter | -0.84 | -0.91 | 0.93 | 0.86 |
| Rhodoplanes | -0.54 | -0.55 | 0.75 | 0.63 |
| Devosia | -0.69 | -0.6 | 0.71 | 0.66 |
| Pseudomonas | -0.69 | -0.63 | 0.87 | 0.85 |
| Mortierella | 0.58 | 0.52 | -0.74 | -0.74 |
| Acremonium | 0.66 | 0.6 | -0.81 | -0.79 |
| Fusarium | 0.78 | 0.69 | -0.91 | -0.83 |
| Rhizophlyctis | -0.68 | -0.59 | 0.9 | 0.85 |
| Basidiobolus | -0.67 | -0.58 | 0.89 | 0.83 |
| Clitopilus | -0.7 | -0.61 | 0.84 | 0.78 |
| Aspergillus | -0.69 | -0.57 | 0.57 | 0.69 |
