# Supplementary material for: Manipulation of the rhizosphere microbial community through application of a new bio-organic fertilizer improves watermelon quality and health
Source: PLoS One. 2018 Feb 16;13(2):e0192967. doi: 10.1371/journal.pone.0192967 (PMC5815603; doi:10.1371/journal.pone.0192967)
Supplement: S6 Table — (DOC) [file pone.0192967.s007.doc]

**S6 Table The correlation between microbial diversity and watermelon quality and disease.**

| **Microbial diversity** | **Disease incidence** | **Disease index** | **Average fruit weight** | **Soluble solids** |
| --- | --- | --- | --- | --- |
| Bacterial Chao 1 | -0.81 | -0.71 | 0.92 | 0.91 |
| Bacterial Shannon | -0.75 | -0.67 | 0.97 | 0.94 |
| Fungal Chao1 | 0.83 | 0.95 | -0.64 | -0.76 |
| Fungal Shannon | 0.69 | 0.48 | -0.94 | -0.88 |
